# ﻿Five new species of the genus *Hermonassa* Walker, 1865 from Xizang Autonomous Region, China (Lepidoptera, Noctuidae, Noctuinae)

**DOI:** 10.3897/zookeys.1179.107587

**Published:** 2023-09-08

**Authors:** Biao Gao, Hui-Lin Han, Vladimir S. Kononenko, Zhao-Hui Pan

**Affiliations:** 1 Northeast Forestry University, School of Forestry, Harbin 150040, China; 2 Northeast Asia Biodiversity Research Center, Northeast Forestry University, Harbin 15004, China; 3 Northeast Forestry University, Ministry of Education, Key Laboratory of Sustainable Forest Ecosystem Management, Harbin 150040, China; 4 Laboratory of Entomology, Federal Scientific Center of the East Asia Terrestrial Biodiversity, Far Eastern Branch, Russian Academy of Sciences, Vladivostok-22, 690022, Russia; 5 Plateau Ecology Research Institute of Tibet Academy of Agriculture and Animal Husbandry, Linzhi 86000, China

**Keywords:** China, *Hermonassa*, new species, Noctuidae, Noctuinae, taxonomy

## Abstract

Five new species of the genus *Hermonassa* Walker, 1865 (*H.nigricans***sp. nov.**, *H.conusa***sp. nov.**, *H.brunneocuprina***sp. nov.**, *H.albimacula***sp. nov.**, and *H.linzhiensis*, **sp. nov.** are described from Autonomic Region Xizang, China (Tibet). *Hermonassanigricans***sp. nov.** is distributed in Nepal and China, and the Himalayan species *H.kalamantra* Kovács, G. Ronkay & L. Ronkay, 2018 is reсorded for China for the first time. The occurrences of *H.anthracina* Boursin, 1967 in Nepal is questionable taking in the account the presence of two externally hardly distinguishable species, *H.kalamantra* and *H.nigricans***sp. nov.** New collecting data for *H.yixincheni* Han & Li, 2007 and *H.oleographa* Hampson, 1911 in China are presented. Five species groups are reviewed, and three species groups are designated.

## ﻿Introduction

The genus *Hermonassa* Walker, 1865 (type species *H.consignata* Walker, 1865, type locality Darjeeling, India) is an exclusively East Asian group of the subfamily Noctuinae with its highest species diversity in the mountains of the Sino-Himalayan subregion in the border between Palaearctic and the Oriental regions. Most species occurs in mountain systems at an altitude higher than 2000 m in Pakistan, India, Nepal and China. Only two northernmost species, *H.arenosa* Butler, 1878 and *H.cecilia* Butler, 1878 occur in the hillsides and low mountains in the south of the Russian Far East, China, Korea, and Japan. To date, more than 90 species of *Hermonassa* are known; among them, 57 of these are recorded in China (Chen, 1999). The history of investigation of the genus in China and East Asia has been described in detail by Han and Li (2007) and Kovács et al. (2018). The numerous literature sources on the genus mainly represent descriptive works. Taking in account the high diversity of the genus and the uniform appearance of species, this group requires basic revision. The global revision of the genus *Hermonassa* was initiated by Kovács et al. (2018).

In the course of the inventory of Chinese Noctuoidea fauna, we investigated the diversity of the Noctuidae in the southeast side of Xizang Autonomic Region (Tibet) from 2010 to 2018. Extensive material on the genus *Hermonassa* in this area enumerates 32 species. Among them, five new species of *Hermonassa* from five species groups are described below. Three species groups are proposed.

## ﻿Materials and methods

The materials for this article were collected in the vicinity of Linzhi City, China at altitudes between 2000 and 6000 meters with a 220V/450W mercury vapour lamp and a DC black light. Standard methods for dissection and preparation of the genitalia slides were used (Kononenko and Han 2007). Moths were photographed using a Nikon D700 camera, whereas the genitalia slides were photographed with an Olympus photo-microscope with the Helicon Focus software and further processed in Adobe Photoshop CC20. The type series of the new species including holotypes are deposited in the collection of Northeast Forestry University (**NEFU**), Harbin, China. The type specimens and other comparative materials were examined from Zoologisches, Forschunginstitut und Museum Alexander Koenig, Bonn, Germany (**ZFMK**), and other museums, listed below and from the literature.

### ﻿Depositories

**HNHM** Hungarian Natural History Museum, Budapest, Hungary Budapest

**MHNG** Muséum d’histoire naturelle, Geneva, Switzerland

**MNHU** Museum für Naturkunde, Humboldt-Universität, Berlin, Germany

**NACRC** National Animal Collection Resource Center, Beijing, China

**NEFU** Northeast Forestry University, Harbin, China

**NHM UK** Natural History Museum (formerly British Museum, Natural History), London, United Kingdom

**NIAES** National Institute of Agro-Environmental Sciences, Tsukuba, Japan

**ZFMK** Zoologisches Forschungsmuseum Alexander Koenig, Bonn

**ZSM**Zoological Museum of the Bavarian State (Zoologische Staatssammlung), München, Germany

## ﻿Taxonomic account

### ﻿Genus Hermonassa

Taxon classificationAnimaliaLepidopteraNoctuidae

Walker, 1865

A8225858-BA3D-597B-BDA5-F47878ED76D2

Hermonassa Walker, 1865, List of the Specimens of Lepidopterous Insects in the Collection of the British Museum 32: 631. Type species: Hermonassaconsignata Walker, 1865 [Darjeeling, India].

### ﻿The *anthracina* species group

The detailed morphological characteristic of the *anthracina* species group was described by Kovács et al. (2018). The most conspicuous characters of this group is the structure of the male genitalia, namely: the bilobate valva, the presence of heavily sclerotised and lateral or proximo-lateral folded projection of the saccular lobe, strongly dilated apically, spatulate uncus, the deltoidal juxta, the very long and ventrally rounded, heavily sclerotised vinculum, and the apically dilated and rounded harpe; the female genitalia have a rather bull-head-shaped, heavily sclerotised antrum and strongly sclerotised quadrangular plates of the ductus bursae. Kovács et al. (2018) listed two species in the group, the Tibetan *H.anthracina* and the southern Himalayan *H.kalamantra*. In the present study, the Tibetan-Himalayan distribution of *H.kalamantra* and Tibetan distribution for *H.anthracina* are clarified; the new species with a Tibetan-Himalayan distribution described here as *H.nigricans* sp. nov. is the third member of the *anthracina* group.

### ﻿ Hermonassa nigricans

Taxon classificationAnimaliaLepidopteraNoctuidae

Gao, Han & Kononenko sp. nov.

89BC1B58-008B-5ECC-A6D3-CAC459469F1A

https://zoobank.org/51C58FF7-BBED-4279-B252-9C3E217B25C8

**Figures 1–10.**
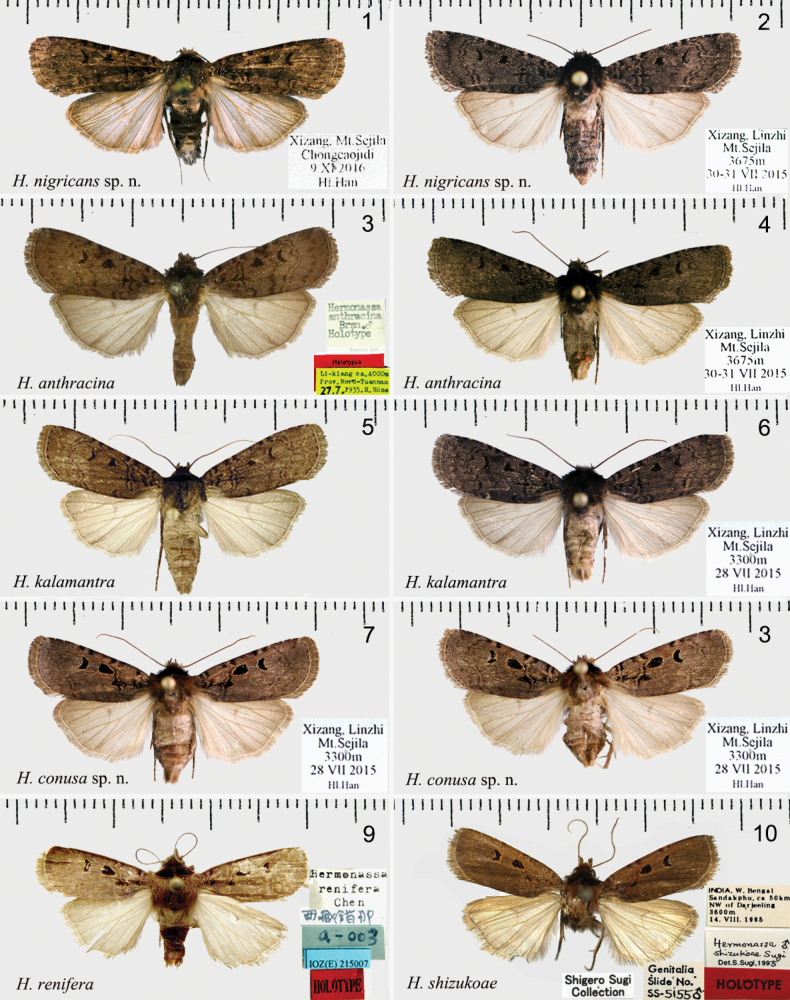
*Hermonassa* spp., adults **1***H.nigricans* sp. nov., ♂, holotype, China **2** Ditto, ♀, paratype **3***H.anthracina*, ♂, holotype, China (ZFMK) **4***H.anthracina*, ♀, China **5***H.kalamantra*, ♂, holotype, Nepal (after Kovács et al. 2018) **6***H.kalamantra*, ♂, China **7***H.conusa* sp. nov., ♂, holotype, China **8** Ditto, ♀, paratype, China **9***H.renifera*, ♂, holotype, China (NACR) **10***H.shizukoae*, ♂, holotype, India (NIAES).

**Figures 11–18.**
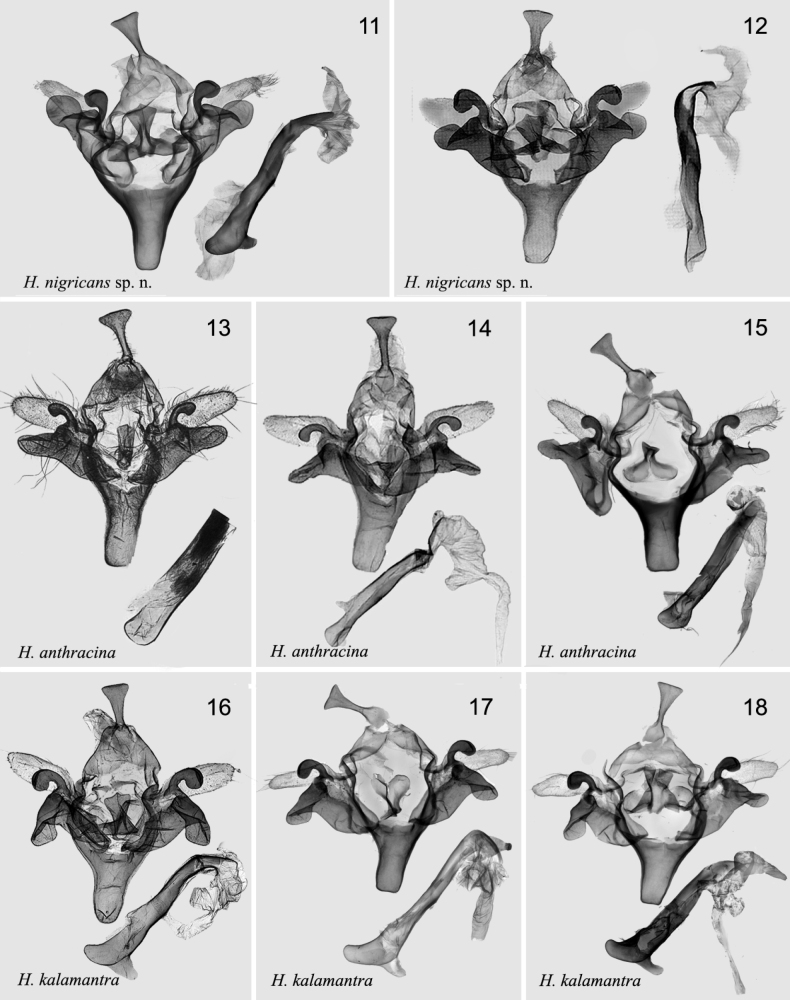
*Hermonassa* spp., male genitalia **11***H.nigricans* sp. nov., holotype, China, genit. prep. GB-101-1 **12***H.nigricans* sp. nov., Nepal (referred as *H.anthracina*, after Sugi, 1995) **13***H.anthracina*, paratype, China (ZFMK); **14***H.anthracina*, China (after Kovács et al. 2018) **15***H.anthracina*, China, genit. prep. hhl-3490-1 **16***H.kalamantra*, paratype, Nepal (after Kovács et al. 2018) **17***H.kalamantra*, China, genit. prep. hhl-3489-1 **18***H.kalamantra*, China, genit. prep. hhl-3488-1.

Figs 1 , 2 , 11 , 12 , 23

Hermonassa anthracina : Sugi 1995: 90, pl. 117, fig. 3, genit. fig. 696 (♂), 732 (♀) (misidentification).

#### Type material.

***Holotype***: ♂, China, Aut. Reg. Xizang, Linzhi City, Mt. Sejila, Cordyceps base, 4121 m, 9 Nov. 2016, H.L. Han leg., genit. prep. GB-101-1. ***Paratypes***: China: 2 ♀, Aut. Reg. Xizang, Linzhi City, Lulang town, Layue village, 2200 m, 14–15 Aug. 2014, H.L. Han leg., genit. prep. GB-20-2, GB-202-2; 1 ♂, 6 ♀, Aut. Reg. Xizang, Linzhi City, Lulang town, Mt. Sejila, Yaguo, 3650 m, 22 Aug. 2014, H.L. Han leg., genit. prep. GB-19-2, GB-26-2, GB-28-1, GB-197-2, GB-200-2, GB-201-2, GB-204-2; 1 ♂, Aut. Reg. Xizang, Linzhi City, Lulang town, Military depot, 3121 m, 3 Aug. 2015, H.L. Han leg., genit. prep. GB-119-1; 1 ♂, Aut. Reg. Xizanzhi City, Lulang town, 3506 m, 5 Aug. 2015, HL Han leg., genit. prep. GB-199-1; 1 ♀, Aut. Reg. Xizang, Linzhi City, Lulang town, Mt. Sejila, Yaguo, 3150 m, 24 Aug. 2015, H.L. Han leg., genit. prep. GB-25-2; 1 ♂, Aut. Reg. Xizang, Linzhi City, Mt. Sejila, Cordyceps base, 4121 m, 9 Nov. 2016, H.L. Han leg., genit. prep. GB-101-1; 1 ♂, Aut. Reg. Xizang, Linzhi City, Zhangmai village, 13 Aug. 2017, H.L. Han leg., genit. prep. GB-120-1; 1 ♀, Aut. Reg. Xizang, Linzhi City, Mt. Sejila, Cordyceps base, 17 Aug. 2017, H.L. Han leg., genit. prep. GB-108-2.

#### Diagnosis.

The new species is superficially very similar to *H.anthracina* (Figs 3, 4, 13–15, 24), and *H.kalamantra* (Figs 5, 6, 16–18, 25) and can barely be distinguished from these species by external characters. The new species is blackish-brown, somewhat darker than both other species; the antemedial line is somewhat broader than in both species, the postmedial line is doubled and more distinct than in both species. The hindwing is somewhat more whitish than in the related species. The main specific differences are in the genitalia of both sexes. In the male genitalia of *H.nigricans* sp. nov. the uncus is more massive and gradually expands from the base to the top (in *H.anthracina* and *kalamantra* it is suddenly expands from 2/3 of the base); the tegumen is broader than in both species; the harpe is more massive and the club-like top of harpe is more swollen compared with *H.anthracina* and *kalamantra*; the sclerotised flattened extension of sacculus is rounded and directed upward (in *H.anthracina* it is directed straight, in *H.kalamantra* downward). The aedeagus of *H.nigricans* sp. nov. is larger than in related species, the carina is broader than in *H.anthracina* and *H.kalamantra*. In the female genitalia: the apophyses posteriores are broader and stronger (in *H.anthracina* and *H.kalamantra* they are slender); the antrum is more massive than in both species, the ductus bursae has two strong sclerotised bands (in *H.anthracina* they are broader and longer, not strongly sclerotised; in *H.kalamantra* they are 2× shorter than in *H.nigricans*).

#### Description.

***Adult*** (Figs 1, 2). Wingspan 30–32 mm. Head dark brown to black; labial palps brown; thorax black to brownish black; abdomen dark brown. Forewing ground colour blackish brown to smoky black; basal line black, double; antemedial line wavy, double, black, distinctly excurved at inner margin; median line very indistinct; postmedial line double, brownish black, excurved; subterminal line wavy and slightly black; terminal line black, dotted line; orbicular spot irregular and deep black; reniform spot crescent-shaped. Hindwing pale greyish white; discal spot arc-shaped, indistinct; vein brown; terminal line same as in forewing; fringe grey. ***Male genitalia*** (Figs 11, 12). Uncus flat, from 1/3 of shovel-shaped base apically. Tegumen broad, ~ 2× the length of uncus. Juxta tongue-shaped anteriorly, large and duck palm-shaped posteriorly. Valva bifurcated, basally broad; sacculus strongly sclerotised, sacculus process flat, tongue-shaped, rounded, with fold; harpe club-shaped, strongly sclerotised, exceed costal margin; cucullus densely setose. Saccus long U-shaped, sclerotised. Aedeagus narrow from caecum to carina and strongly sclerotised; caecum curved, with knife-shaped process at dorsal part. Vesica membranous, multiple median diverticula extending with a small cornutus. ***Female genitalia*** (Fig. 23). Papillae anales broad, slightly sclerotised. Apophyses anteriores ~ 1/4× the length of apophyses posteriores. Ostium bursae broad, flat, antevaginal plate concave, strongly sclerotised. Ductus bursae flat, moderately sclerotised, with two strongly sclerotised irregular bands. Appendix bursae slightly sclerotised, wrinkled. Corpus bursae long, ~ 4× length of ductus bursae, with longitudinal wrinkles, and two slender longitudinal signa bands.

#### Etymology.

The name of the new species is derived from the Latin word *nigricans* that means black.

#### Distribution and biology.

*Hermonassanigricans* is distributed in southwest China (Aut. Reg. Xizang) and Nepal. The species is rather common in the grassland and shrubby areas in eastern spurs of the Tibet plateau (Aut. Reg. Xizang) at altitude 2200–4100 m. It occurs sympatrically with its related species *H.anthracina* and *H.kalamantra.* Collecting period range from early August to beginning of November.

#### Remark.

The species was reported from Nepal due to a misidentification; the adult and its male genitalia were illustrated by Sugi (1995) as *H.anthracina* (reproduced in Fig. 12).

### ﻿ Hermonassa anthracina

Taxon classificationAnimaliaLepidopteraNoctuidae

Boursin, 1967

F47BCF2E-22DB-50D8-9288-BE81305D8E68

Figs 3 , 4 , 13–15 , 24

Hermonassa anthracina Boursin, 1967, Zeitschrift der Wiener Entomologischen Gesellschaft 52: 26, pl. 1, fig. 4; Poole 1989: 502; Chen et al. 1991: 92, pl. 4, fig. 8, genit. fig. 53, male; Krusek and Behounek 1996a, pl. 50, fig. 8; 1996b: pl. 80, fig. 1; Kovács et al. 2018: 298, pl. 1, figs 1–4, genit. figs 1, 2.

#### Type material.

***Holotype***: ♂, [China] yellow label: Li-Kiang, ca 4000 m, Prov. North Yuennan, 27.7.1935, H. Hӧne /red label Holotypus/ white label *Hermonassaanthracina* Brsn. ♂ Boursin det./. Deposited in ZFMK, Bonn, Germany, examined.

#### Other material examined.

China, 2 ♀, Aut. Reg. Xizang, Linzhi City, Lulang town, Mt. Sejila, 3650 m,22 Aug. 2014, H.L. Han leg., genit. prep. GB-26-2, GB-197-2; 1 ♀, Aut. Reg. Xizang, Linzhi City, Mt. Sejila, 4713 m, 12–13 Aug. 2014, H.L. Han leg., genit. prep. GB-198-2; 1 ♂, Aut. Reg. Xizang, Linzhi City, Mt. Sejila, 3675 m, 30–31 July 2015, H.L. Han leg., genit. prep. hhl-3490-1; 1 ♂, Aut. Reg. Xizang, Linzhi City, Lulang town, 5 Aug. 2015, H.L. Han leg., genit. prep. GB-199-1; 1 ♀, 1 ♂, Prov. Qinghai, Maixiu nursery, 14 July 2020, H.L. Han & J. Wu leg., genit. prep. hhl-4741-1, hhl-4742-2 (coll. NEFU).

#### Distribution and biology.

*Hermonassaanthracina* is known in China from the provinces of Qinghai, Yunnan, Sichuan, and Aut. Reg. Xizang in southwest China. Adults occur in the rocky slope meadows in coniferous forest at altitudes of 2000–4700 m. The flight period is between mid-July and mid-August.

#### Remarks.

Boursin (1967) listed numerous paratypes collected in Nepal (coll. ZSM), south Tibet, and north India (coll NHM) in the description of H.anthracina; however. Kovács et al. (2018) did not refer to the material of *H.anthracina* from Nepal, but they listed numerous specimens of this species from China. They noted that “the type series [of *H.anthracina* in Boursin’s (1967) description] is mixed, the paratypes from Nepal and northern India representing in fact the southern Himalayan sister species *H.kalamantra* sp. nov.” The record of *H.anthracina* from Nepal (Sugi 1995) belongs to *H.nigricans* sp. nov. (see above). Therefore, taking in the account the presence of three externally similar species in Tibetan-Himalayan subregion the question about the presence of *H.anthracina* in Nepal and north India remains open.

### ﻿ Hermonassa kalamantra

Taxon classificationAnimaliaLepidopteraNoctuidae

Kovács, G. Ronkay & L. Ronkay, 2018

7324CE36-255A-563D-A0EA-69CB01CEF871

Figs 5 , 6 , 16–18 , 25

Hermonassa kalamantra Kovács, G. Ronkay & L. Ronkay, 2018, Revue suisse de Zoologie 126 (2): 298, pl. 1, figs 5–8, genitalia figs 3–5.

#### Type material.

***Holotype***: ♂, Nepal, Annapurna Himal, Mesokantu pass, 4200 m. Deposited in the collection of HNHM, Budapest, Hungary (not examined).

#### Other material examined.

China, 2 ♂, Aut. Reg. Xizang, Linzhi City, Mt. Sejila, 3300 m, 28 July 2015, H.L. Han leg., genit. prep. hhl-3488-1, hhl-3489-1 (coll. NEFU).

#### Distribution and biology.

*Hermonassakalamantra* occurs in Nepal and the southwest China (Aut. Reg. Xizang). Judging from the collecting data in Nepal it is a common species at altitudes of 2000–4000 m; adults flying from May to September (Kovács et al. 2018). In Aut. Reg. Xizang it is known from only two specimens and appears rare compared with *H.anthracina* and *H.nigricans* sp. nov. adults have been collected in late July.

#### Remark.

The species is reported for China for the first time.

##### ﻿The *dispila* species group

The members of the *dispila* species group are externally similar to taxa of the *anthracina* group, while some species have narrower forewing and different wing colouration. As mentioned by Kovács et al. (2018: 301) they are morphologically more heterogeneous and diverse: “The genitalia of these species show different trends of change in certain features which led to the often remarkable differences between the male genital capsule of the different lineages of the clade. Due to the basically uniform external appearance and the genital features of the entire species group, as well as partly overlapping differential characters, the taxa of the species group can be arranged into different lineages.” Those authors listed 12 species in the *dispila* species group; however, the exact number of species and selection of the main lineages of this group are likely will be clarified in the course of further revisions.

### ﻿ Hermonassa conusa

Taxon classificationAnimaliaLepidopteraNoctuidae

Gao, Han & Kononenko sp. nov.

A12C7C7A-B7CA-5D31-8948-15776E3319ED

https://zoobank.org/A737AEAE-94E9-43AA-8F51-18DC02AA63FD

**Figures 19–27.**
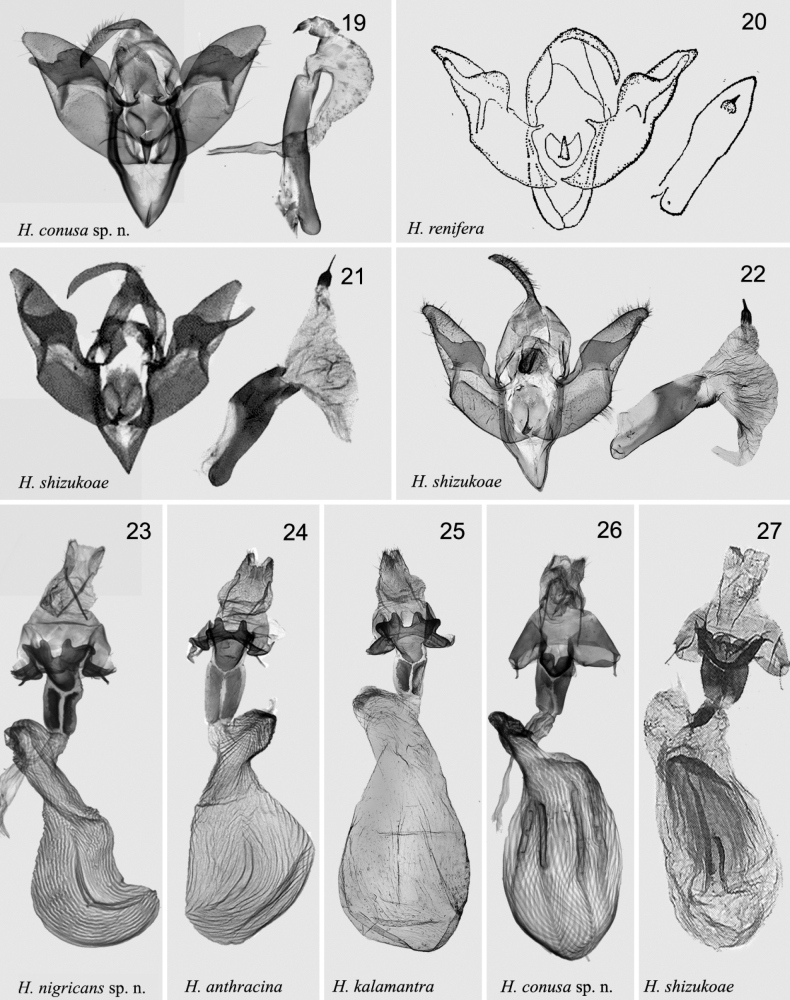
*Hermonassa* spp., male (19-22) and female (23-27) genitalia **19***H.conusa* sp. nov., holotype, genit. prep. hhl-3492-1 **20***H.renifera*, paratype (after Chen 1991) **21***H.shizukoae*, holotype, India (after Sugi 1995) **22***H.shizukoae*, Nepal (after Kovács et al. 2018) **23***H.nigricans*, paratype, genit. prep. hhl-3493-2 **24***H.anthracina*, China, (after Kovács et al. 2018) **25***H.kalamantra*, Nepal (after Kovács et al. 2018) **26***H.conusa* sp. nov., paratype, China, genit. prep. hhl-3491-2 **27***H.shizukoae*, paratype, India (after Sugi 1995).

Figs 7 , 8 , 19 , 26

#### Type material.

***Holotype***: ♂, China, Aut. Reg. Xizang, Linzhi City, Mt. Sejila, 3300 m, 28 July 2015, H.L. Han leg., genit. prep. hhl-3492-1. ***Paratypes***: China: 1 ♂, Aut. Reg. Xizang, Linzhi City, Mt. Sejila, 3650 m, 22 Aug. 2014, H.L. Han leg., genit. prep. GB-66-1; 1 ♂, 2 ♀, Aut. Reg. Xizang, Linzhi City, Mt. Sejila, 3300 m, 28 July 2015, H.L. Han leg., genit. prep. hhl-3491-2; 1 ♂, Aut. Reg. Xizang, Linzhi City, Mt. Sejila, Yaguo, 3150 m, 24 Aug. 2015, H.L. Han leg., genit. prep. GB-29-1 (coll. NEFU).

#### Diagnosis.

*Hermonassaconusa* sp. nov. (Figs 7, 8, 19, 26) together with *H.renifera* (Figs 9, 20) and *H.shizukoae* (Figs 10, 21, 22, 27) are the most similar species in the *dispila* species group. Externally *H.conusa* differs from related species by more a robust habit, a broader forewing shape, dark brown with pale violet tint colouration of the forewing (in *H.renifera* and *H.shizukoae* the forewing colouration is brown with a reddish tint, especially in the costal part) and by a more coarse forewing pattern with more distinct basal, ante-, and postmedial lines (they are weakly expressed in *H.renifera* and *H.shizukoae*); more distinct claviform, and larger drop-shaped orbicular than in related species (in *H.renifera* and *H.shizukoae* the claviform is weekly expressed and the orbicular is small drop-shaped). The male genitalia of *H.conusa* sp. nov. differ from those of the related species by shorter tegumen (it is ~ 1/3× height of vinculum vs ~ 1/2× in *H.renifera* and *H.shizukoae*), the broad valva with prominent subbasal extensions of the costa (in the related species the valva are much narrower, without extension of the costa); the harpe with a large and broad base, which reaches but does not exceed the dorsal margin of valva (in the related species the base of harpe is narrower and exceed the dorsal margin of valva); the top of harpe is short, straight and broad (vs long, narrow and curved in *H.renifera* and *H.shizukoae*). The juxta is wide, rectangular, with a medium process, inverted nail-shaped (in *H.renifera* and *H.shizukoae* the juxta is tongue-shaped, or nearly heart-shaped with a smaller and shorter process); the sacculus is much broader than in related species. The aedeagus is much longer and narrower than in *H.renifera* and *H.shizukoae*; the cornutus much smaller and thinner; the vesica is tube-like, with smaller subbasal diverticulum compared with *H.renifera* and *H.shizukoae.* In the female genitalia, antevaginal plate is narrow and nearly heart-shaped vs broad and calyculate in *H.shizukoae*; ductus bursae is long and slightly sclerotised vs short in *H.shizukoae*; corpus bursae is shorter, with four band-like signa (in *H.shizukoae* corpus bursae is longer and the signa are slighter and wrinkle-like.

#### Description.

***Adult*** (Figs 7, 8). Wingspan 29–31 mm. Head, labial palps, thorax dark brown, patagia and tegulae black; abdomen black, mixed with greyish white at segments 1–3, abdominal tuft distinct. Forewing ground colour dark brown; basal line double, black; antemedial line double, black, slender, curved, slightly wavy; median line indistinct; postmedial line double, brown, mixed with black, excurved; subterminal line indistinct; terminal line greyish white, slender; orbicular spot drop-shaped and reniform spot crescent-shaped, both with pale brown edges. Hindwing pale, light greyish brown; discal spot arc-shaped; veins brown; fringe pale brown. ***Male genitalia*** (Fig. 19). Uncus slightly hooked apically. Tegumen broad, slightly longer than uncus, ~ 1/3× vinculum length. Juxta rectangular with medial inverted nail-shaped process. Valva broad medially, gradually narrower apically; cucullus flat, blunt round; harpe slightly drumstick-shaped, with straight top, extending to ventral margin of valva. Saccus broad, V-shaped, strongly sclerotised. Aedeagus straight; caecum narrow ~ 1/7× aedeagus length; carina long and thin serrated; vesica membranous, with short thin cornutus. ***Female genitalia*** (Fig. 26). Papillae anales broad, slightly sclerotised. Apophyses anteriores slender, ~ 1/3× length of apophyses posteriores. Ostium flat, antevaginal plate almost rounded-triangular with central narrow cut, strongly sclerotised. Ductus bursae broad, flat, with anterior half partially sclerotised, with two irregular plates. Corpus bursae long and swollen, ~ 6× length of ductus bursae, densely wrinkled, with four slender signa bands of different in lengths.

#### Etymology.

The species name refers to the large, sclerotised, conical extension on the base of the juxta in the male genitalia of the new species.

#### Distribution and biology.

*Hermonassaconusa* is known only from Linzhi mountain range in Aut. Reg. Xizang, in southwest China. The species occurs in grassland meadows in the coniferous forest at an altitudinal range of 3150–3650 m. Adults have been collected between the end of July and the end of August.

### ﻿ Hermonassa renifera

Taxon classificationAnimaliaLepidopteraNoctuidae

Chen, 1991

0CF2C7F0-8762-504E-A92A-9793BC625CD4

Figs 9 , 20

Hermonassa renifera Chen, 1991, Acta entomologica Sinica 34(3): 353, fig. 2; Hreblay and Ronkay 1998: 133 (senior synonym of H.shizukoae Sugi, 1995); Kovács et al. 2018: 306 (distinct species).

#### Type material.

***Holotype***: ♂, China, Aut. Reg. Xizang, Medog, 2000 m. Deposited in Institute of Zoology, Academia Sinica, Beijing, adult examined.

#### Distribution and biology.

The species is known only from its type locality, Aut. Reg. Xizang, China.

#### Remarks.

Hreblay and Ronkay (1998) considered this taxon conspecific with *H.shizukoae* but latter small differences were found in the details of the male genitalia and Kovács et al. (2018) concluded that *H.renifera* and *H.shizukoae* are two distinct species. The species was described from two specimens, and no additional material was ever collected. Because of the holotype of *H.renifera* was not dissected, the ink drawing of the male genitalia probably belongs to the paratype. Here we accept Kovács et al. (2018) point of view, but further study of this species pair on the basis of newly collected materials is necessary.

### ﻿ Hermonassa shizukoae

Taxon classificationAnimaliaLepidopteraNoctuidae

Sugi, 1995

979210C1-BC8F-564C-AF70-1370A203BB94

Figs 10 , 21 , 22 , 27

Hermonassa shizukoae Sugi, 1995, Tinea 14, (suppl. 2): 92, pl. 117, fig. 17; Hreblay and Ronkay 1998: 133 (junior synonym of H.renifera Chen, 1991); Kovács et al. 2018: 306, pl. 3, figs 5–8, genit. figs 15, 16 (distinct species).

#### Type material.

***Holotype***: ♂, India, West Bengal, Sandadakphu, ca 50 km NW of Darjiling, 3600 m, 14.viii.1985(W.Thomas), genitalia slide No SS-5155 “Shigero Sugi Collection”, “HOLOTYPE” (red label), *Hermonassashizukoae* Sugi ♂, det. S. Sugi, 1995” (coll. NIAES), examined.

#### Distribution and biology.

The species is distributed in north India and Nepal where it is rather common at altitude ca 3600 m (Kovács et al. 2018).

#### Remarks.

The species is the southern Himalayan representative of *H.renifera* - *H.shizukoae* species pair (Kovács et al. 2018).

##### ﻿The *cuprina* species group

The *cuprina* species group includes two species, *H.cuprina* and *H.brunneocuprina* sp. nov. This species group can be characterised by the uniform external appearance with a relatively large size, the narrow forewings, cupper-brown or brown colouration and by the forewing pattern with more or less clearly expressed thin basal, antemedial, and postmedial transverse lines and the presence of black contrasting claviform, and the orbicular and reniform stigmata. The main diagnostic features of the male genitalia are the relatively short, flat and apically dilated, spatulate uncus; juxta small, three leaved, dart-shaped, large U-shaped vinculum, the relatively narrow simple valva, thin, upcurved harpe, and the slightly curved aedeagus with a sclerotised comb in the carina. The female genitalia are characterised by the deeply split antevaginal plate, deep cup-like antrum, a thin, medium length ductus seminalis, and the presence of two longitudinal signa on the corpus bursae.

### ﻿ Hermonassa brunneocuprina

Taxon classificationAnimaliaLepidopteraNoctuidae

Gao, Han & Kononenko sp. nov.

C35019C5-98BA-5743-A6BB-115BABC14030

https://zoobank.org/190F476A-E4D0-451A-9800-FBA0FEAED1F3

**Figures 28–34.**
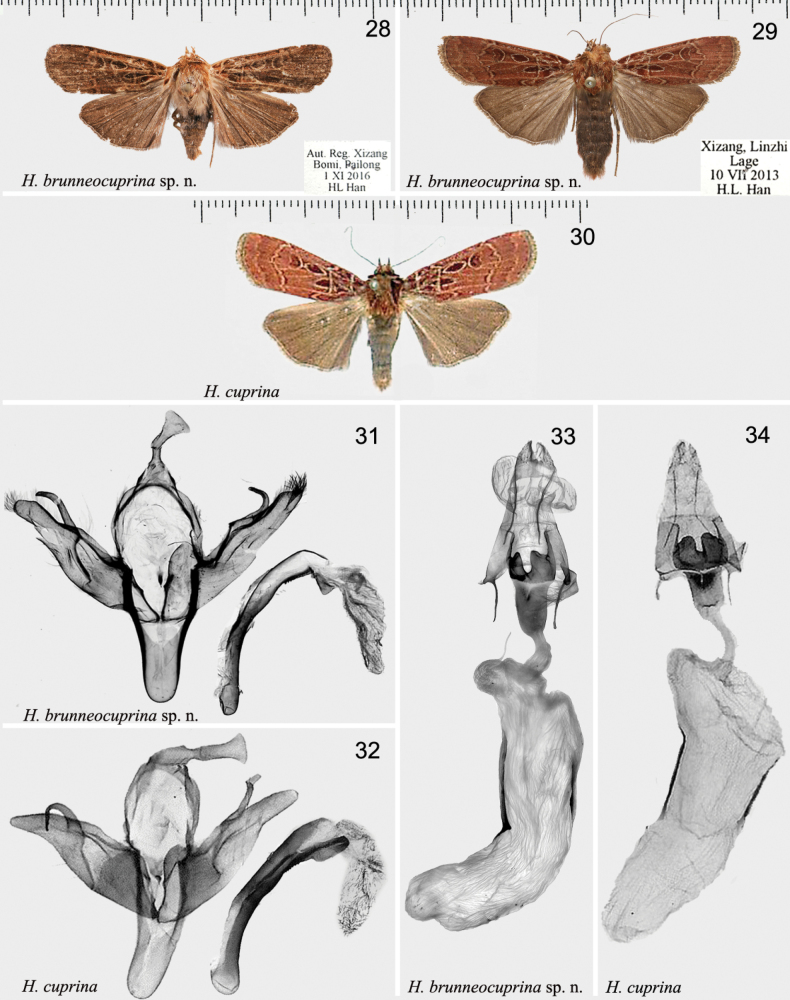
*Hermonassa* spp., adults (28-30), male (31-32) and female (33-34) genitalia **28***H.brunneocuprina* sp. nov., ♂, holotype **29** Ditto, ♀, paratype **30***H.cuprina*¸ Nepal (after Sugi 1995) **31***H.brunneocuprina* sp. nov., holotype, genit. prep. GB-283-1 **32***H.cuprina* (after Sugi 1995) **33***H.brunneocuprina* sp. nov., paratype, genit. prep. GB-322-2 **34***H.cuprina*, India (after Sugi 1995).

Figs 28 , 29 , 31 , 33

#### Type material.

***Holotype***: ♂, China, Aut. Reg. Xizang, Linzhi City, Bomi County, Pailong Countryside, 2005 m, 1 Nov. 2016, H.L. Han leg., genit. prep. GB-283-1. ***Paratypes***: 2 ♀, China, Aut. Reg. Xizang, Linzhi City, Lage, 10 July 2013, H.L. Han leg., genit. prep. GB-321-2, GB-322-2; 3 ♂, 2 ♀, Aut. Reg. Xizang, Linzhi City, Bomi County Pailong Countryside, 1 Nov. 2016, H.L. Han leg., genit. prep. GB-282-2; 1 ♀, Aut. Reg. Xizang, Linzhi City, Motuo County, 6200 m, 22–24 July 2017, S.Y. Huang leg., genit. prep. GB-295-2; 1 ♀, Prov. Yunnan, Tengchong City, Jietou town, Shahe, 6 Nov. 2020, J. Wu et al. leg., genit. prep. GB-294-2; 1 ♀, Prov. Yunnan, Tengchong City, Jietou town, Shanxintou, 8 Nov. 2020, J. Wu et al. leg., genit. prep. GB-296-2 (coll. NEFU).

#### Diagnosis.

This new species is superficially similar to *H.cuprina* (see Figs 30, 32, 34), but can be distinguished from the latter one by the following characters. In the adult: the basal, antemedial and postmedial lines with double yellowish brown lines (in *H.cuprina* they are whitish); the subterminal line is thin (in *H.cuprina* it is distinct); the terminal line is solid (in *H.cuprina* it is as dotted line); the orbicular spot is short and drop-shaped (in *H.cuprina* it is long). In the male genitalia: the top of the uncus is large, shovel-shaped apically; the uncus is smaller in the new species (in *H.cuprina* it is somewhat larger again); the cucullus is rounded apically (in *H.cuprina* it is tapering); the juxta is three leaved dart-shape (in *H.cuprina* it is morning glory-shaped); the carina process is small and triangular (in *H.cuprina* it is large, band-shaped). In the female genitalia: the 8^th^ segment has a swollen membranous sac (in *H.cuprina* it is absent); the antevaginal plate is concave (in *H.cuprina* it is less concave); ductus bursae is relatively broad (in *H.cuprina* it is narrow); corpus bursae is elongated S-shaped (in *H.cuprina* it is curved medially only).

#### Description.

***Adult*** (Figs 28, 29). Wingspan 39–41 mm. Head reddish brown; labial palps more orange; thorax reddish brown, patagia with white central spots; abdomen dark reddish brown, abdominal tuft distinct. Forewing ground colour dark reddish brown; transverse double lines with pale brown inside; basal line black, dotted; antemedial line black, bending at Sc+R_1_, incurved to 2A, then excurved inwardly; median line indistinct; postmedial line wavy, dark brown mixed with black; subterminal line thin; terminal line solid, thin; orbicular spot drop-shaped, framed with yellow; reniform spot short, broad bean-shaped, edged with pale scales; terminal area somewhat paler than ground colour. Hindwing dark brown; discal spot weak, arc-shaped; vein reddish brown; fringe grey. ***Male genitalia*** (Fig. 31). Uncus medium long, sclerotised, flat, shovel-shaped in apical 1/3. Tegumen wide, as long as uncus. Juxta three leaved, dart-shaped. Valva broad medially, gradually narrower to apex; cucullus bluntly round; left harpe smooth, incurved (genitalia slide slightly fractured), right one excurved; sacculus same width basally in left valva, in right valva narrower. Saccus long, U-shaped. Aedeagus upcurved, slightly wavy; caecum ~ 1/8× length of aedeagus; carina serrated, with sclerotised medium triangle-shaped process; vesica membranous, densely covered with small cornuti dorsally. ***Female genitalia*** (Fig. 33). Papillae anales short, slightly sclerotised. The 8^th^ segment with swollen membranous sac. Apophyses slender and long, anteriores apophyses ~ 1/2× length of posteriores. Ostium broad and flat, antevaginal plate concave-shaped, with deep cut medially, strongly sclerotised. Ductus bursae short, tubular. Corpus bursae very long, S-shaped, ~ 4× length of ductus bursae; corpus bursae slightly sclerotised anteriorly, wrinkled, bearing two strongly sclerotised signa bands, right signum ~ 2/3× length of left one.

#### Etymology.

The name of the new species refers to its deep brown wing colouration compared with copper-brown colouration of its related species *H.cuprina*.

#### Distribution and biology.

*Hermonassabrunneocuprina* is known only from Linzhi mountain range in Aut. Reg. Xizang, the Southwest China. The species occurs in grassland meadows in coniferous forest at altitude range 2000–6200 m. Adults have been collected in early November.

### ﻿ Hermonassa cuprina

Taxon classificationAnimaliaLepidopteraNoctuidae

Moore, 1882

C0854133-FC92-5C6C-8C7B-4FA428762C5B

Figs 30 , 32 , 34

Hermonassa cuprina Moore, 1882, Descriptions of new Indian Lepidopterous Insects from the Collection of the Late Mr. W.S. Atkinson. Heterocera (continued) (Cymatophoridae-Herminiidae). Part II: 120; Hampson 1903: 357; Boursin 1967: 36; Poole, 1989: 502; Sugi 1995: 90, pl. 117, fig. 1, genit. fig. 691(♂), 721 (♀); Krusek and Behounek 1996a: pl. 50, fig. 2.

#### Type material.

***Syntype(s)***: India, Prov. West Bengal, Darjeeling, Deposited in NHM London, United Kingdom and MNHU, Berlin, Germany), not examined.

#### Distribution and biology.

North India: Prov. Sikkim, Prov. West Bengal, Darjiling [Darjeeling].

#### Remarks.

*Hermonassacuprina* has not yet been found in China. The syntypes of this species are deposited in NHM UK and MNHU (Moore 1882; Poole 1989). The lectotype is not designated. For the illustration of *H.cuprina* (Figs 30, 32, 34) we follow the treatment and illustrations in Sugi (1995) of this species from north India.

##### ﻿The *dictyota* species group

The *dictyota* species group externally can be characterized by the relatively large size (wingspan 38–40 mm), the robust habitus and the reticulate forewing pattern atypical for most *Hermonassa* spp. The male genitalia of these species are rather uniform and characterized by having an apically swollen or spatulate uncus, anchor-like, apically hooked juxta, moderate harpe, variable in length saccular extension and the presence of a pollex at the apical part of the valva. In the female genitalia, the antevaginal plate is large, with a deep cut in the centre, a sclerotised antrum, the relatively short ductus bursae and the sclerotised proximal part of the corpus bursae. The *dictyota* species group comprises the following four species: *H.dictyota*, *H.legraini*, *H.yixincheni*, and *H.albimacula* sp. nov.

### ﻿ Hermonassa albimacula

Taxon classificationAnimaliaLepidopteraNoctuidae

Pan, Han & Kononenko sp. nov.

A744CCAD-D11C-52AC-8611-11C4A4339159

https://zoobank.org/E3F148AA-23FF-4D2F-A9C9-F5090CE2889E

**Figures 35–42.**
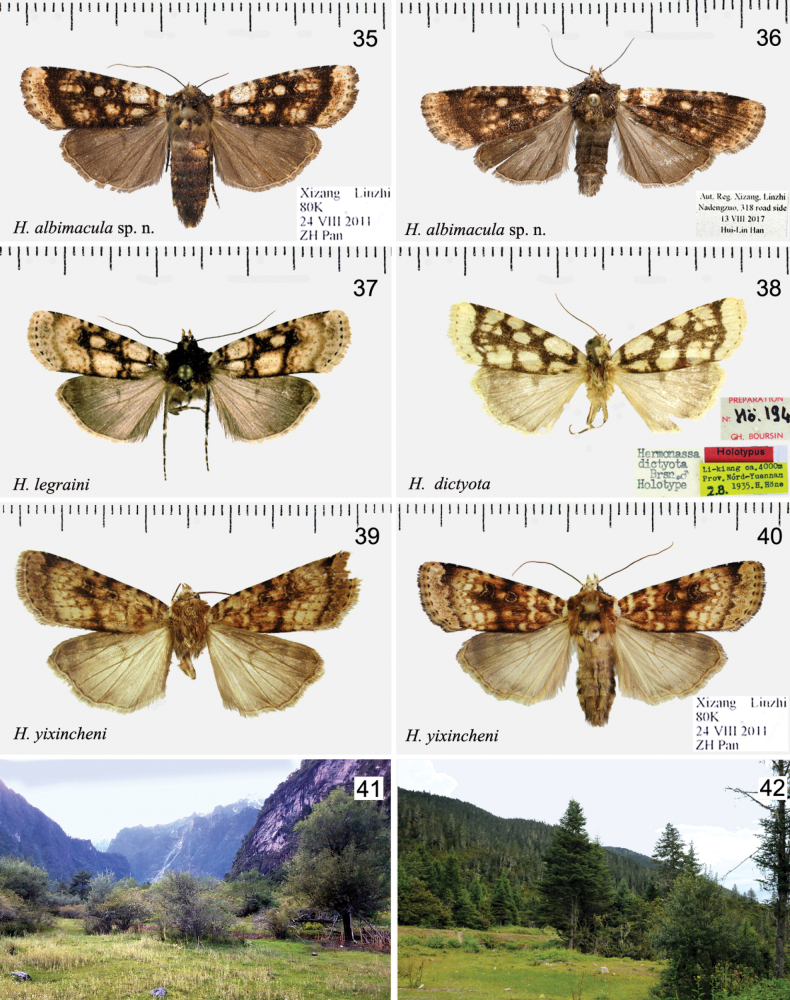
*Hermonassa* spp., adults (**35–40**) and habitats (**41, 42**) **35***H.albimacula* sp. nov., ♀, holotype, China **36** Ditto, ♂, paratype, China **37***H.legraini*, ♂, Taiwan (after Ronkay et al. 2013) **38***H.dictyota* ♂, holotype, China (ZFMK) **39***H.yixincheni*, ♂, holotype, China **40** Ditto, China, ♀ **41** Habitat of *H.albimacula* sp. nov., vic. Linzhi City, Guxiang, 3000 m **42** Habitat of *H.linzhiensis* sp. nov., vic. Linzhi City, Mt. Sejila, 3500 m.

Figs 35 , 36 , 51 , 60

#### Type material.

***Holotype***: ♀. China, Aut. Reg. Xizang, Linzhi City, 3000 m, 23 Aug. 2011, Z.H. Pan leg., genit. prep. hhl-5284-2. ***Paratypes***: China: 2 ♀, Prov. Sichuan, Hailuo Valley, 30 July 2003, M. Wang et al. leg., genit. prep. GB-79-2, GB-80-2; 1 ♀, Aut. Reg. Xizang, Linzhi City, 30 Aug. 2001, Z.H. Pan leg.; 1 ♀, Aut. Reg. Xizang, Linzhi City, 3000 m, 1–3 Aug. 2011, Z.H. Pan leg.; 2 ♀, Aut. Reg. Xizang, Linzhi City, 3000 m, 24 Aug. 2011, Z.H. Pan leg.; 1 ♂, Aut. Reg. Xizang, Linzhi City, Nadengzuo, 17 Aug. 2014, H.L. Han leg.; 1 ♀, Aut. Reg. Xizang, Linzhi City, Guxiang, 2970 m,14–19 July 2017, W. Da leg., genit. prep. GB-97-2; 1 ♀, Aut. Reg. Xizang, Linzhi City, Bomi County, Xuyu, 20 Sept. 2016, Z.H. Pan leg., genit. prep. GB-103-2; 1 ♀, Aut. Reg. Xizang, Linzhi City, Bomi County, Shuangyu, 7 Aug. 2017, H.L. Han leg.; 2 ♂, Aut. Reg. Xizang, Linzhi City, Nadengzuo, 13 Aug. 2017, H.L. Han leg., genit. prep. GB-118-1, GB-318-1; 2 ♀, Aut. Reg. Xizang, Linzhi City, Lulang town, 15 Aug. 2017, H.L. Han leg., genit. prep. GB-157-2, GB-191-2 (coll. NEFU); 2 ♀, China, West Sichuan, road Yaan/Kangding, Erlang Shan Mt., H–2161 m, 29°87'340"N, 102°30'970"E, 11–12 Sept. 2017, Saldaitis leg, slide GYP 4845, coll. P. Gyulai, Miskolc, Hungary.

#### Diagnosis.

*Hermonassaalbimacula* sp. nov. is superficially similar to *H.legraini* (Fig. 37) and *H.dictyota* (Fig. 38), but differs from both by a darker reddish brown ground colour of the forewing, a broader subterminal line, and the less expressed pale elements of the wing pattern. The male genitalia differs from those of *H.legraini* by the thinner uncus, the shape of juxta with a shorter apical extension, the shape of the valva, shorter and more tapered than in *H.legraini*; the harpe and saccular extension are ~ 2× shorter than in *H.legraini*; the pollex is smaller, but broader and placed more apically compared with *H.legraini.* The female genitalia of the new species differs from those of *H.legraini* by a broader antrum with broad antevaginal plate deeply cut in the centre, the 3× broader ductus bursae, sclerotised apically and the shape of the bursae with a broad cervix.

#### Description.

***Adult*** (Figs 35, 36). Wingspan 39–41 mm. Head white, labial palps dark brown; thorax dark brown; abdomen dark reddish brown, mixed with dark grey, tuft indistinct. Forewing ground colour dark reddish brown; transverse line double, with white in room; basal line dark brown to brown; antemedial line dark brown to brown, slightly curved; median line blackish brown to brown, broad, wavy; postmedial line dark brown, slender, excurved; subterminal line broad, black band-like; terminal line dotted; orbicular spot black, small, partly edged with pale line; reniform spot broad bean-shaped, partly edged with pale. Hindwing dark brown mixed with grey; discal spot, indistinct; terminal line yellowish white; fringe brown. ***Male genitalia*** (Fig. 51). Uncus curved basally, broader apically. Tegumen wide, ~ 1.2× length of uncus. Juxta incurved anchor-shaped, with long thin, hooked apically central process. Valva relatively narrow, straight, slightly curved apically; cucullus bluntly tapered; saccular process long, slightly wavy, reach pollex; harpe thin, relatively short, curved basally, reach costal margin, pollex short, placed in apical fourth of valva. Saccus long, narrow V-shaped. Aedeagus slightly wavy, with long, narrow serrated carina; vesica membranous, with a strong spine-like cornutus. ***Female genitalia*** (Fig. 60). Papillae anales short, slightly sclerotised. Apophyses thin, long; apophyses anteriores ~ 1/2× of length of apophyses posteriores. Ostium broad and slightly wrinkled, antevaginal plate strongly sclerotised, concave, with deep central cut. Ductus bursae long, broad, strongly sclerotised anteriorly. Corpus bursae 2× longer than ductus bursae, wrinkled; with four long signa bands.

#### Etymology.

The species name refers to forewing pattern of the new species. It formed from the Latin words *albus* (white) and *macula* (a spot).

#### Distribution and biology

(Fig. 41). *Hermonassaalbimacula* is known from Linzhi mountain range in Aut. Reg. Xizang, four specimens were collected in Prov. Sichuan, southwest China. The species occurs in the grassland shrubby meadows bordered with cliffs at altitude 2900–3000 m. Adults were collected from the end of July to late September.

### ﻿ Hermonassa legraini

Taxon classificationAnimaliaLepidopteraNoctuidae

Plante, 1994

75AA3761-8B94-59CE-A8AC-6E098577CA51

Figs 37 , 52 , 61

Hermonassa legraini Plante, 1994, Tyo-to-Ga 44(4): 226, figs 3 (adult), 10 (male genitalia); Hreblay and Ronkay 1997: 25, fig. 126; Ronkay et al. 2013: 33, pl. 8: figs. 1, 2, pl. 27, figs 37-40, gen. figs 57, 58; Fu et al. 2013: 518, pl. 45, fig.10.

#### Type material.

***Holotype***: ♂, Taiwan, Prov. Tayuling, Hualien Co., 30 km SE of Lishan, 2'650 m, 25.ix.1992, F. Aulombard & J. Plante. MHNG ENTO-12727. Dissected, ex slide J. Plante 1557, not examined.

#### Distribution and biology.

According to Ronkay et al. (2013) and Fu et al. (2013), *H.legraini* is endemic to Taiwan: “One of the rarest Noctuidae species in Taiwan. It is confined to the higher mountains in Central Taiwan between 1950–3100 m altitudes. The adults can be found from late July to October. Univoltine” (Fu et al. 2013: 518).

### ﻿ Hermonassa dictyota

Taxon classificationAnimaliaLepidopteraNoctuidae

Boursin, 1967

8C6DDC96-43A7-5234-BC7C-5F7A3CE09673

Figs 38 , 54

Hermonassa dictyota Boursin, 1967, Zeitschrift der Wiener Entomologischen Gesellschaft 52: 28, pl. 1: 8 ♀ (paratype), pl. 5, fig. 8 (♂, holotype); Poole 1989: 503; Chen et al. 1991: 97, pl. 4, fig. 12; Krusek and Behounek 1996b: pl. 80, fig 3.

#### Type material.

***Holotype***: ♂, [China] yellow label: Li-Kiang, ca 4000 m, Prov. Nord Yuennan, 2.8.1935, H. Hӧne/ red label: Holotypus/ white label *Hermonassadictyota* Brsn. ♂. Boursin det./ Preparation No194 CH. Boursin/. Deposited in ZFMK, Bonn, Germany, examined.

#### Distribution and biology.

*Hermonassadictyota* is known only from its type locality, Likiang, Prov. Yunnan, southwest China. The holotype and several paratypes of both sexes were collected from beginning of August to mid- September 1935 at altitude 2000–4000 m.

### ﻿ Hermonassa yixincheni

Taxon classificationAnimaliaLepidopteraNoctuidae

Han & Li, 2007

6BFBFEFC-F13E-556E-A97F-CD971969FB1C

Figs 39 , 40 , 53 , 62

Hermonassa yixincheni Han & Li, 2007, Journal of Asia-Pacific Entomology 10(3): 193, figs 1–4.

#### Type material.

***Holotype***: ♂, ca 2100 m, Hanmi army depot, Motuo, Prov. Xizang, China, 18–27 VIII 2005 (leg. H. Huang, D. Zhou, L. Tang), slide No. HHL-1009, examined. ***Paratypes***: 3♂, 1♀, same data as holotype, female genitalia slide No. HHL-1115. Holotype and paratypes are deposited in NEFU, Harbin.

#### Other material examined.

5 ♂, 2 ♀, Aut. Reg. Xizang, Linzhi City, 24 Aug. 2011, Z.H. Pan leg., genit. prep. GB-71-2, GB-72-1, GB-238-1, hhl-5282-2; 1 ♂, Xizang, Linzhi City, Motuo County, Guxiang, 2970 m, 14–19 July 2017, W. Da, leg. (coll. NEFU).

#### Distribution and biology.

*Hermonassayixincheni* is known only from Linzhi mountain range in Aut. Reg. Xizang, southwest China. The species occurs in grassland meadows in the coniferous forest at altitude range 2100–2970 m. Adults have been collected from mid-July to late August.

##### ﻿The *pallidula* species group

The *pallidula* species group is close to the *dictyota* group. Externally it differs from most other *Hermonassa* spp. by relatively large size (wingspan 38–40 mm), rather broad forewing shape and pale yellowish brown colour of forewing with weak main elements of the Noctuinae pattern and contrast black or dark brown orbicular and reniform stigmata. The male genitalia of the species of *pallidula* group are similar to those of the *dictyota* species group. The *pallidula* species group comprises following taxa: *H.pallidula*, *H.hoenei*, *H.ellenae* (with the subspecies *H.e.ellenae*, *H.e.tapaishana*, and *H.e.robusta*), and *H.albimacula* sp. nov.

### ﻿ Hermonassa linzhiensis

Taxon classificationAnimaliaLepidopteraNoctuidae

Pan, Han & Kononenko sp. nov.

74D0AF84-8547-5C37-A8BF-6E783824E6B9

https://zoobank.org/58092DA0-5C5A-4CCB-AA1B-22BF90D12AD7

**Figures 43–50.**
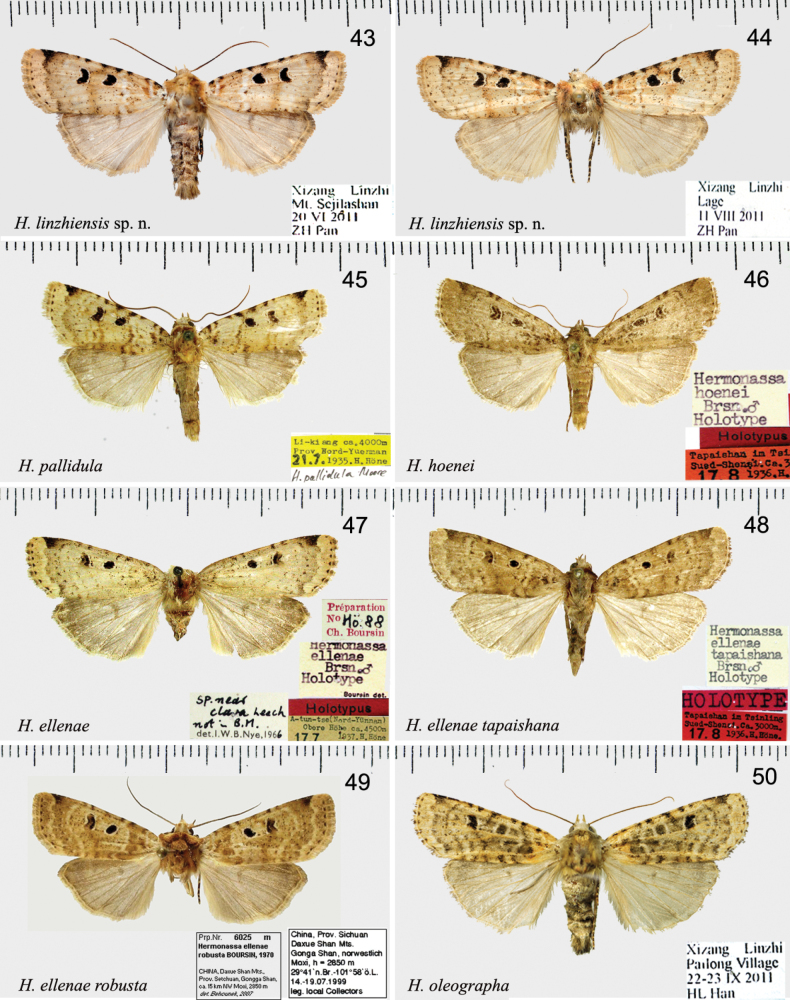
*Hermonassa* spp., adults, males **43***H.linzhiensis* sp. nov., ♂, holotype **44** Ditto, ♀, paratype **45***H.pallidula* (ZFMK) **46***H.hoenei*, holotype (ZFMK) **47***H.ellenae*, holotype (ZFMK) **48***H.ellenaetapaishana*, holotype (ZFMK) **49***H.ellenaerobusta*, authentic specimen (ZSM) **50***H.oleographa*.

Figs 43 , 44 , 55 , 63

#### Type material.

***Holotype***: ♂. China, Aut. Reg. Xizang, Linzhi City, Mt. Sejila, 20 June 2011, Z.H. Pan leg., genit. prep. hhl-5278-1. ***Paratypes***: 2 ♂, 1 ♀, Aut. Reg. Xizang, Linzhi City, Mt. Sejila, 20 June 2011, Z.H. Pan leg., genit. prep. GB-316-1, hhl-5279-2, GB-235-1; 1 ♀, Aut. Reg. Xizang, Linzhi City, Lage, 11 Aug. 2011, Z.H. Pan leg., genit. prep. GB-68-2; 1 ♀, Aut. Reg. Xizang, Linzhi City, Mt. Sejila, 27 Aug. 2012, H.L. Han leg., genit. prep. GB-116-1; 2 ♂, Aut. Reg. Xizang, Linzhi City, Hanmi, 2200 m, 20–28 July 2013, Z.H. Pan leg., genit. prep. GB-234-1; 2 ♂, 1 ♀, Aut. Reg. Xizang, Linzhi City, Lulang Army Station, 26 July 2013, H.L. Han leg., genit. prep. hhl-5281-2; 2 ♂, 1 ♀, Aut. Reg. Xizang, Linzhi City, Mt. Sejila, 27 July 2013, H.L. Han leg., genit. prep. GB-183-1, hhl-5280-1; 2 ♂, 2 ♀, Aut. Reg. Xizang, Linzhi City, Mt. Sejila, 4050 m, 4 Aug. 2013, H.L. Han leg., genit. prep. GB-102-1, GB-187-1; 1 ♂, 1 ♀, Aut. Reg. Xizang, Linzhi City, Lulang town, 5 Aug. 2015, H.L. Han leg., genit. prep. GB-67-1; 18 ♂, 2 ♀, Aut. Reg. Xizang, Linzhi City, Mt. Sejila, 3500 m, 16 Aug. 2014, H.L. Han leg., genit. prep. GB-233-1; 2 ♂, 3 ♀, Aut. Reg. Xizang, Linzhi City, Lulang Station, 15 Aug. 2017, H.L. Han, leg. genit. prep. GB-112-1, GB-158-2, GB-159-2 (coll. NEFU).

#### Diagnosis.

*Hermonassalinzhiensis* sp. nov. is superficially similar to *H.pallidula* (Fig. 45), *H.ellenae* (Figs 47–49), and *H.hoenei* (Fig. 46). It differs from these related species by relatively larger size (wingspan 34–48 mm), more stout body, and with pale reddish tint of the pale brownish yellow forewing. The forewing pattern differs from the similar species by more strongly expressed basal, ante-, and postmedial lines, the somewhat larger and more contrasting orbicular and reniform spots and clearly bean-like shape of the reniform spot. In the male genitalia *H.linzhiensis* sp. nov. (Fig. 55) is most similar to *H.pallidula* (Fig. 56), but differs from the latter by somewhat narrower uncus, broader valva, larger and sharp pollex, somewhat broader harpe, and basal extension of sacculus. Aedeagus narrower than in *H.pallidula*, the single cornutus smaller.

#### Description.

***Adult*** (Figs 43, 44). Wingspan 34–38 mm. Head pale beige; labial palps earthy yellow; thorax pale beige, patagia beige, tegulae with three white central spots; abdomen brown, tuft distinctly marked. Forewing ground colour yellowish white, mixed with pale yellow, with pale reddish tint; transverse line single; basal line white; antemedial line greyish white, wavy; median line slightly reddish brown, broad, diffused, curved; postmedial line reddish brown, thin, wavy; subterminal line, reddish brown, waved, thinner, only with broad dark dot at costal area; terminal line formed by small dark brown dots; orbicular spot broad, slightly drop-shaped, edged with white; reniform short, narrow bean-shaped (or C-shaped), edged with white. Hindwing whitish, mixed with pale yellow; discal spot arched, indistinct; vein pale brown; fringe pale yellow; costal margin covered with brown scales. ***Male genitalia*** (Fig. 55). Uncus rather large, diamond-shaped apically. Tegumen broad, as long as uncus. Juxta strongly sclerotised anchor like, with hook-shaped apical extension. Valva broad; harpe long, thin, curved. Saccular process moderate, nearly reaching harpe base; pollex broad, relatively long, sharp; cucullus short, blunt apically. Saccus strongly sclerotised thin, elongated V-shaped. Aedeagus straight, caecum ~ 1/4× length of aedeagus, carina serrated; vesica membranous with strong cornutus on broad base at middle of ventral side. ***Female genitalia*** (Fig. 63). Papillae anales short, slightly sclerotised. Apophyses thin and short, anteriores apophyses ~ 1/2× length of posteriores ones. Ostium broad and flat, antevaginal plate strongly sclerotised, concave with quadrangular cut in centre. Ductus bursae strongly sclerotised. Corpus bursae long, sack-shaped, ~ 2.5× length of ductus bursae, with strongly sclerotised, anteriorly wrinkled cervix.

#### Etymology.

The name is derived from the species’ type locality, the vicinity of Linzhi City, Aut. Reg. Xizang, China.

#### Distribution and biology

(Fig. 42). *Hermonassalinzhiensis* is known only from Linzhi mountain range in Aut. Reg. Xizang, southwest China. The species occurs in grassland meadows in the dense coniferous forest at an altitude range of 2200–3500 m. Adults have been collected from early June to mid-August.

### ﻿ Hermonassa pallidula

Taxon classificationAnimaliaLepidopteraNoctuidae

(Leech, 1900)

D424E35B-CFC4-54DB-A14B-9D3EF4FAED5C

Figs 45 , 56

Graphiphora pallidula Leech, 1900, Transactions of the Entomological Society of London 1900: 39; Hampson 1903: 362, pl. 68, fig. 24; Jordan and Warren 1909–1914: 57, pl. 15k; Corti and Draudt 1931–1938: 62, fig. 62, pl. 8d; Kozhanchikov 1937: 266; Boursin 1967: pl. 4: 6; Poole 1989: 503; Chen 1982: 2049; Chen et al. 1991: 93, pl. 4:26, ♂ genit. fig. 54; Krusek and Behounek 1996a: pl. 51, fig. 4; 1996b: pl. 79: 4, pl. 80, fig. 5.

#### Type material.

***Syntypes***: 1♂, 2♀, “W China, Omei-shan” [China, Prov. Sichuan], deposited in NHM (Beccaloni et al. 2003), not examined.

#### Other material examined.

2 ♂, specimens, identified by Ch. Boursin, “Likiang, ca 4000 m, Nord Yuennan, 21 July 1935, H. Hӧne”/. Deposited in the coll. ZFMK.

**Figures 51–56.**
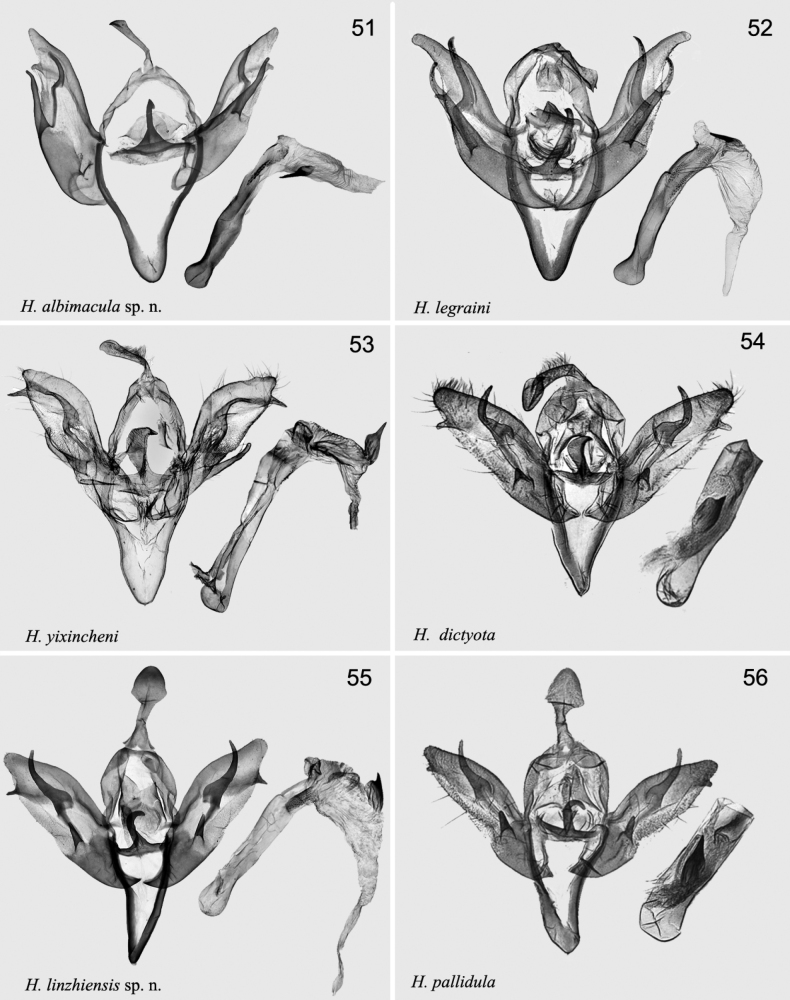
*Hermonassa* spp., male genitalia **51***H.albimacula* sp. nov., paratype, China, genit. prep. GB-318-1 **52***H.legraini*, Taiwan (after Ronkay et al. 2013) **53***H.yixincheni*, holotype, China, genit. prep. hhl-1009-1 **54***H.dictyota*, holotype, China, (ZFMK) **55***H.linzhiensis* sp. nov., holotype, China, genit. prep. hhl-5278-1 **56***H.pallidula*, China (after Boursin 1967).

#### Distribution and biology.

The species is known from its type locality, Omeishan, Sichuan Prov., and from Likiang, Yunnan Prov., southwest China; the male genitalia were illustrated by Boursin (1967). The specimens from Likiang were collected 21 July 1935 at an altitude 4000 m.

### ﻿ Hermonassa hoenei

Taxon classificationAnimaliaLepidopteraNoctuidae

Boursin, 1967

CC8E6178-A552-5409-8373-5B8F2C3C712F

Figs 46 , 57

Hermonassa hoenei Boursin, 1967. Zeitschrift der Wiener Entomologischen Gesellschaft 52: 28, pl. 1, fig. 7, pl. 5. fig. 7; Poole 1989: 503; Krusek and Behounek 1996a: pl. 51, fig. 5; 1996b: pl. 80, fig. 4.

#### Type material.

***Holotype***: ♂ [China] red label: Tapaishan im Tsinling Sued Shensi, ca 3000 m, 17.8.1935 H. Hӧne/ red label: Holotypus/ white label: *Hermonassahoenei* Brsn. ♂, Holotype/. Deposited in ZFMK, Bonn, examined.

#### Distribution and biology.

*Hermonassahoenei* is known only from its type locality, Tapaishan, Tsinling Mts, Prov. Shaanxi, China. The holotype and ca 20 paratypes were collected in same locality at altitudes of 1700 m and 3000 m in August 1936 (Boursin 1967).

### ﻿ Hermonassa ellenae ellenae

Taxon classificationAnimaliaLepidopteraNoctuidae

Boursin, 1967

76A7BEBC-C2AB-5E89-886B-6BC3E1419AB4

Figs 47 , 58

Hermonassa ellenae Boursin, 1967, Zeitschrift der Wiener Entomologischen Gesellschaft 52: 27, pl. 1, fig. 5; Boursin 1967: 27, pl. 1, fig 56, genit. fig. 4:5; 1970: 47, Abb. 56, 57; Poole 1989: 503 (subsp.); Krusek and Behounek 1996b: pl. 79, fig. 12, pl. 80, fig 67.

#### Type material.

***Holotype***: ♂ [China] brownish label: A-tun-tse, Nord Yuennan, Obere Hӧhe 4500 m, 17.7.1937 H. Hӧne/ red label: Holotypus/ white label: *Hermonassaellenae* Brsn. ♂, Holotype/. Deposited in ZFMK, Bonn, examined.

#### Distribution and biology.

*Hermonassaellenaeellenae* is known only from its type locality, A-tun-tse, Prov. Yunnan, China. The holotype was collected 17 July 1937 at an altitude of 3000 m (Boursin 1967).

### ﻿ Hermonassa ellenae tapaishana

Taxon classificationAnimaliaLepidopteraNoctuidae

Boursin, 1967

05664D64-0501-54C3-A7EA-7ACCC6A6F7DF

Fig. 48

Hermonassa ellenae tapaishana Boursin, 1967, Zeitschrift der Wiener Entomologischen Gesellschaft 52: 27, pl. 1, fig. 6; Poole 1989: 504 (subsp.); Krusek and Behounek 1996b: pl. 79, fig. 1, pl. 80, fig 6.

#### Type material.

***Holotype***: ♂ [China] red label: Tapaishan im Tsinling, Sued Shensi, ca 3000 m, 17.8.1935 H. Hӧne/ red label: Holotype/ white label: *Hermonassaellenaetapaishana* Brsn. ♂, Holotype/. Deposited in ZFMK, examined.

**Figures 57–63.**
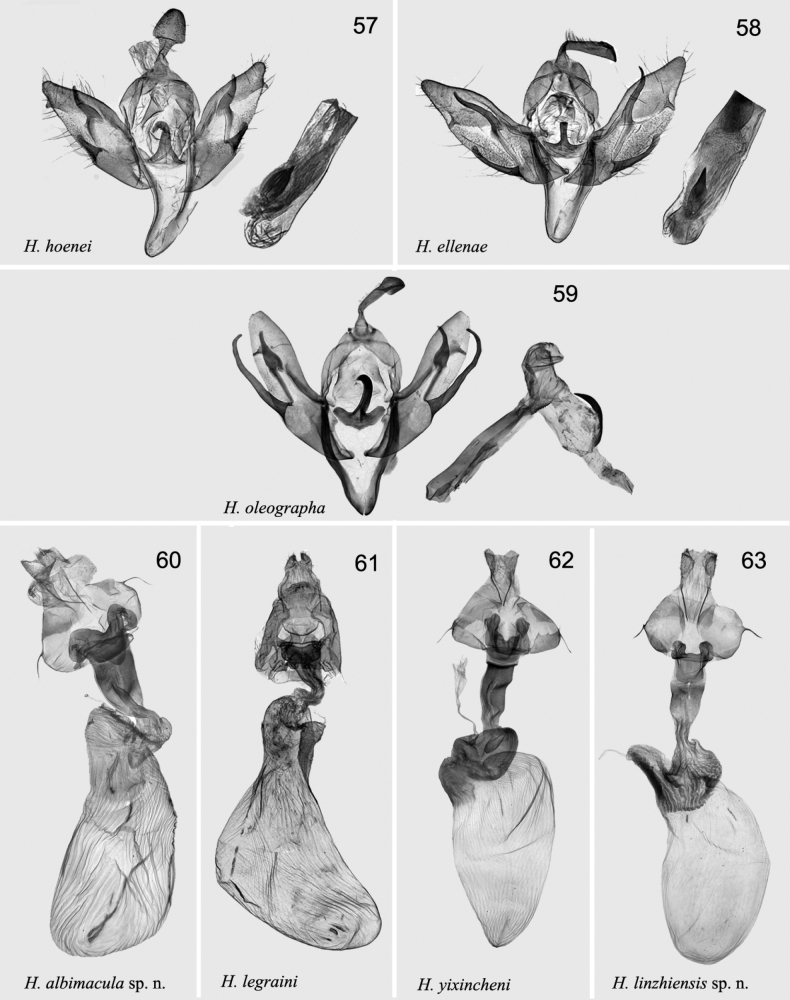
*Hermonassa* spp., male (57-59) and female (60-63) genitalia **57***H.hoenei*, paratype, China (ZFMK) **58***H.e.ellenae*, holotype, China, (ZFMK) **59***H.oleographa*, China, genit. prep. hhl-5283-1 **60***H.albimacula* sp. nov., paratype, China, genit. prep. hhl-5284-2 **61***H.legraini*, Taiwan (after Ronkay et al. 2013) **62***H.yixincheni*, paratype, China, genit. prep. hhl-5282-2 **63***H.linzhiensis* sp. nov., paratype, China, prep. hhl-5279-2.

#### Distribution and biology.

*Hermonassaellenaetapaishana* is known only from its type locality, Tapaishan, Tsinling Mts, Prov. Shaanxi, China. The holotype was collected 17 August 1936 at an altitude of 3000 m. Except for the holotype, Boursin (1967) mentioned ca 20 specimens of both sexes, all collected in the same locality in July – August 1936.

#### Remark.

As mentioned by Boursin (1967) the male genitalia of *H.ellenaetapaishana* are identical with those of *H.ellenaeellenae*.

### ﻿ Hermonassa ellenae robusta

Taxon classificationAnimaliaLepidopteraNoctuidae

Boursin, 1970

121A1D49-DA96-5A09-88EF-77F895873BAF

Fig. 49

Hermonassa ellenae robusta Boursin, 1970, Entomops, Nice 18: 47, Abb. 56, 57 (adult); Poole 1989: 503 (subsp.).

#### Type material.

***Holotype***: ♂, South West China, Sitchuan, Kukal-a-shan. Deposited in ZSM, München, not examined.

#### Other material examined.

1 ♂, China, Prov. Sichuan, Daxue Shan Mts, Gonga Shan, NW Moxi, H= 2850 m, 29º41' n. Br 101º58' o.L 14–19 July 07.1999, leg. local Collector / Prp. Nr. 6025 *Hermonassaellenaerobusta* BOURSIN, 1970, China, Prov. Sichuan, Gonga Shan, 15 km NW Moxi, 2850 m, det. Behounek, 2007.

#### Distribution and biology.

The subspecies *H.ellenaerobusta* is known from the Prov. Sichuan, southwest China in Kukal-a-shan and Gonga ranges at an altitude 2850 m.

#### Remarks.

The subspecies *H.ellenaerobusta* was described by male (holotype) and a female (paratype) from Prov. Sichuan (coll. ZSM). In the description of this taxon Boursin (1970) mentioned that the male genitalia are identical with other subspecies of *H.ellenae*. The male specimen from the collection of Mr. G. Behounek (Fig. 49) and its genitalia slide were compared with the type specimen of *H.ellenaerobusta* and its genitalia preparation and found to be of the same species.

### ﻿ Hermonassa oleographa

Taxon classificationAnimaliaLepidopteraNoctuidae

Hampson, 1911

38596118-EC18-58EB-9B71-5C3AA473C31E

Figs 50 , 59

Hermonassa oleographa Hampson, 1911, Annals and Magazine of Natural History (8)8: 416; Boursin 1967: 67; Poole 1989: 503; Plante 1994: 227; Yoshimoto 1994: 99, pl. 83, fig. 17; Sugi 1995: 90, pl. 117, fig. 4, genit. fig. 697 ♂; Krusek and Behounek 1996a: pl. 51, fig. 7.
Hermonassa griseosignata Chen, 1983, Acta entomologica Sinica 26(3):334, fig. 1; Poole 1989: 503; Chen et al. 1991: 94, pl. 4, fig. 24.

#### Type material.

***Syntypes***: *H.oleographa*: [India] Sikkim, NHM [BMNH], London (not examined). ***Holotype***: *H.griseosignata* ♂ China, Xizang, Cona Magmang, 2900 m, 6 Aug. 1974, examined.

#### Other material examined.

China, 1 ♂, Aut. Reg. Xizang, Linzhi City, 1–30 July 2009, Z.H. Pan leg.; 1 ♂, Reg. Xizang, Linzhi City, 24 Aug. 2011, Z.H. Pan leg.; 5 ♂, Aut. Reg. Xizang, Linzhi City, Bomi County, Pailong Countryside, 22–23 Sept. 2011, H.L. Han leg., hhl-5283-1; 6 ♂, Aut. Reg. Xizang, Linzhi City, Bomi County, Pailong Countryside, 13 Sept. 2012, Z.H. Pan leg., genit. prep. GB-69-1, GB-70-1 (coll. NEFU).

#### Distribution and biology.

The species is distributed in north India (Sikkim), Nepal, Bhutan, and southwest China. In total 13 males were collected in the vicinity of Linzhi City, Prov. Xizang at altitudes of 2000–3000 m.

## Supplementary Material

XML Treatment for Hermonassa

XML Treatment for Hermonassa nigricans

XML Treatment for Hermonassa anthracina

XML Treatment for Hermonassa kalamantra

XML Treatment for Hermonassa conusa

XML Treatment for Hermonassa renifera

XML Treatment for Hermonassa shizukoae

XML Treatment for Hermonassa brunneocuprina

XML Treatment for Hermonassa cuprina

XML Treatment for Hermonassa albimacula

XML Treatment for Hermonassa legraini

XML Treatment for Hermonassa dictyota

XML Treatment for Hermonassa yixincheni

XML Treatment for Hermonassa linzhiensis

XML Treatment for Hermonassa pallidula

XML Treatment for Hermonassa hoenei

XML Treatment for Hermonassa ellenae ellenae

XML Treatment for Hermonassa ellenae tapaishana

XML Treatment for Hermonassa ellenae robusta

XML Treatment for Hermonassa oleographa

## ﻿Additional information

### Author ORCIDs

Hui-Lin Han https://orcid.org/0000-0002-2045-6182

Vladimir S. Kononenko https://orcid.org/0000-0001-6103-4800

### Data availability

All of the data that support the findings of this study are available in the main text.
